# Membranous or membranous‐like GN: A case report of massive proteinuria, positive serum with negative PLA2R on biopsy

**DOI:** 10.1002/ccr3.1849

**Published:** 2018-10-09

**Authors:** Gurwant Kaur, Guoli Chen

**Affiliations:** ^1^ Department of Medicine (Nephrology) Penn State Milton S. Hershey Medical Centre Hershey Pennsylvania; ^2^ Department of Pathology Penn State Milton S. Hershey Medical Centre Hershey Pennsylvania

**Keywords:** membranous glomerulonephritis, membranous‐like glomerulonephritis, nephrotic syndrome, PLA2R, spikes, weak immunoglobulin deposition for IgG

## Abstract

This case report represents primary membranous glomerulonephritis (MGN) with positive serum anti‐PLA2R antibodies, 2+ positivity for IgG4 on immunofluorescence with routine fresh‐frozen sections and negative PLA2R stain on biopsy. He was treated as primary MGN based on positive serum PLA2R and the absence of clinical symptoms or signs suggestive of any secondary MGN.

## 1 INTRODUCTION

Sixty‐five‐year‐old male with nephrotic syndrome underwent renal biopsy which showed features of membranous glomerulonephritis (MGN) on light and electron microscopy. He had positive serum anti‐PLA2R antibodies. Immunofluorescence revealed 2+ IgG4 and was negative for anti‐PLA2R. Membranous‐like glomerulonephritis was considered in differential along with membranous GN.

Nephrotic syndrome is bread and butter for nephrology referral. It is defined as proteinuria >3.5 g/dL, edema, hypoalbuminemia, hyperlipidemia, and hyperlipiduria. MGN is one of the major causes of nephrotic syndrome. Predominant immunoglobulin (Ig) G granular pattern is seen in MGN with subclass IgG4 in idiopathic and non‐IgG4 subclass is prevalent in secondary MGN.^1^ C1q is suggestive of secondary MGN.^1^ We present a case showing features of membranous nephropathy, such as thick basement membrane and spike formation but showed only weak deposition of immunoglobulins (Ig) in the setting of nephrotic syndrome with 14 g of proteinuria. Amount of proteinuria was massive and disproportionate to the strength of positivity of IgG on immunofluorescence (IF). Positive anti‐PLA2R antibodies though they were checked 3 weeks after being on treatment helped point toward primary MGN.

## 2 CASE REPORT

Sixty‐five‐year‐old Caucasian male with coronary artery disease with a left anterior descending artery stent placed about 4 years ago after an abnormal nuclear stress and hypertension for 15 years was referred to nephrology clinic. He was recently discharged from emergency department for leg swelling, positive blood, and more than 300 mg of proteins in urine. He had seen a cardiologist in mean time and evaluation was unremarkable. His presenting weight was 120 kg, body temperature was 36.5°C, his pulse was 52 beats/min and regular, and his blood pressure was 150/98 mm Hg. Physical examination was pertinent for bilateral lower leg edema up to thighs. He was on atenolol, aspirin, irbesartan (300 mg daily), and furosemide. He was up to date for age‐appropriate health screening.

## 3 LABORATORY DATA

On laboratory data, he was found to have 8 g of proteinuria on spot urine protein and creatinine ratio and it was 14 g on 24‐hour urine collection. Urine had small hemoglobin, 0‐4 RBC. Serum creatinine was 0.85 mg/dL, C3 was 112 mg/dL, C4 was 17 mg/dL, uric acid was 6.7 mg/dL, hemoglobin was 13.0 g/dL, platelets were 171 K/µL, and hepatitis B and C were negative. He had marked low serum proteins of 6.5 g/dL and serum albumin of 2 g/dL. His ANCA and ANA were negative. Renal ultrasound was unremarkable with right kidney measuring 13.5 cm and the left kidney measuring 14.6 cm. His latest echocardiogram showed 60% ejection fraction. He underwent a renal biopsy to evaluate his nephrotic syndrome.

### 3.1 Light microscopy (LM)

**Figure 1:**
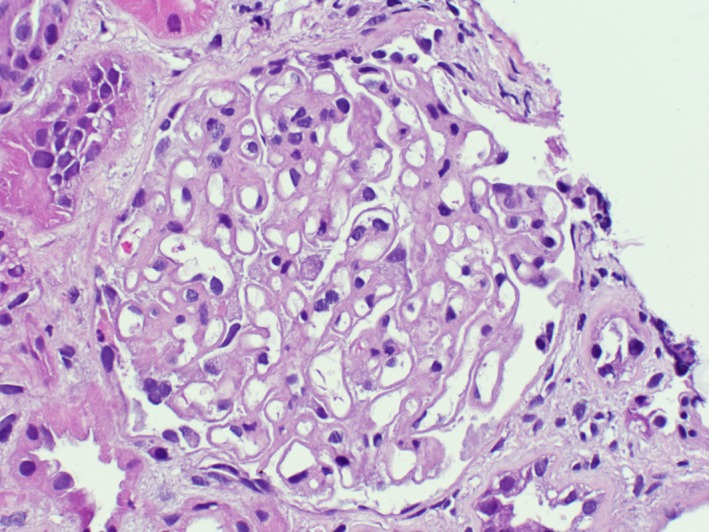
Light microscopy showing diffusely and globally thickened capillary walls. There was mild mesangial expansion, but no significant proliferation was noted

**Figure 2:**
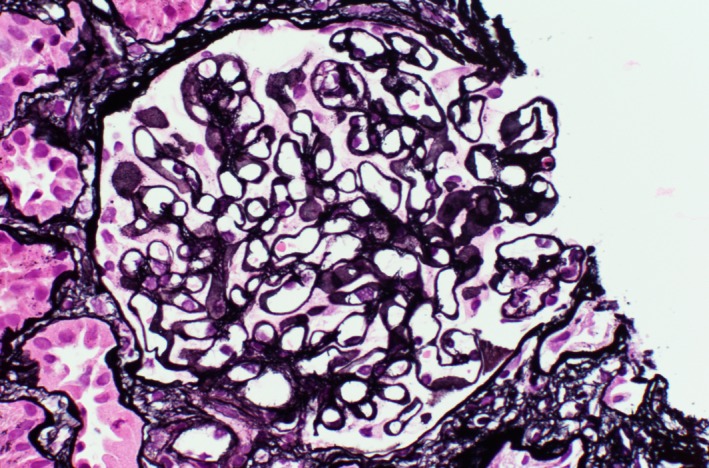
Thickened glomerular basement membrane by Jones methenamine silver stain (original magnification ×400)

Two cores of predominantly renal cortex with 25 glomeruli were reviewed. Three of which were globally sclerotic. The capillary walls were diffusely and globally thickened (Figures 1 and 2) with segmental spikes. There was mild mesangial expansion, but no significant proliferation was noted. No crescents, necrosis, or interstitial inflammation was noted. It had mild interstitial fibrosis and tubular atrophy. Six arteries were noted; some had mild‐to‐moderate atherosclerosis. No definitive arteriolar hyalinosis was noted.

### 3.2 Immunofluorescence

Multiple glomeruli were reviewed on fresh‐frozen section. Only patchy, mild staining of IgG (1+), C3 (1+), albumin (0‐1+), kappa (0‐1+), and lambda (0‐1+) light chains were noted in the capillary walls of glomeruli. No significant staining IgA or IgM was noted. Lambda and kappa chains stained intratubular casts equally. Positive and negative controls were stained appropriately. The glomeruli showed 2+ granular capillary loop staining with IgG4. IgG1, IgG2, and IgG3 were negative. IF staining for PLA2R was negative in glomeruli.

### 3.3 Electron microscopy (EM)

**Figure 3:**
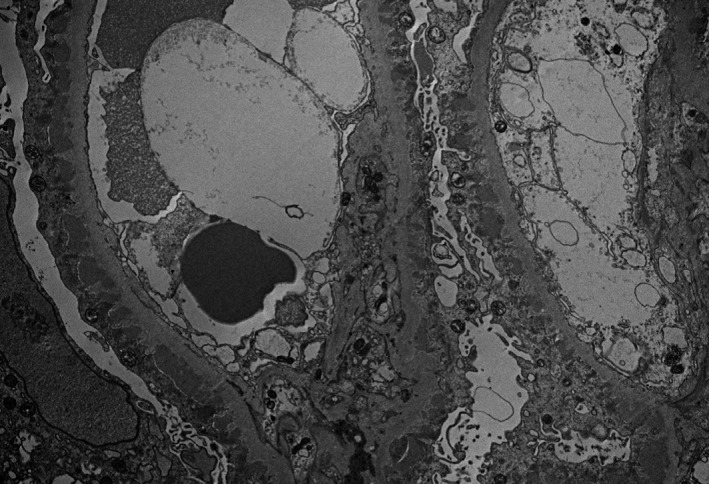
Electron‐dense deposits—numerous subepithelial and intramembranous deposits are present (unstained, original magnification ×12 000)

**Figure 4:**
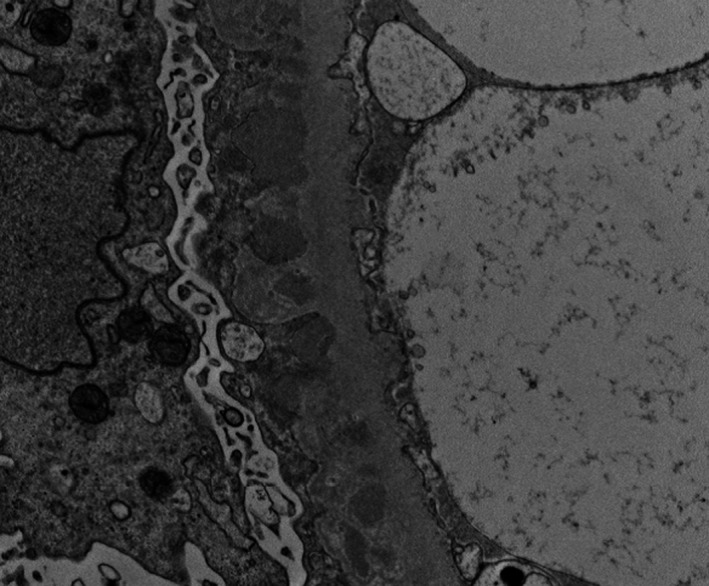
Electron microscopy—numerous subepithelial and intramembranous deposits are present (unstained, original magnification ×12 000)

Extensive subepithelial and focally intramembranous electron densities (Figures 3 and 4) were noted along with extensive foot process effacement on the capillary surface.

### 3.4 Clinical course

He was started on prednisone and was continued on maximum dose of angiotensin receptor blocker (irbesartan). Within 3 weeks of steroids along with continuation of maximum dose of Angiotensin II Receptor Blockers (ARBs), he responded very well with improvement in proteinuria from 14 g to only 4 g. His renal function remained stable and lost 20 pounds during this time. In the meantime, his anti‐PLA2R antibodies came as mild positive (23 RU/mL), making the diagnosis of primary membranous GN though they were checked after him being on steroids for 3 weeks. It is possible that he might have had higher levels of anti‐PLA2R antibodies if were checked at the time of initial presentation. It helped in confirming the diagnosis of primary MGN. He has been tapered off steroids and has been doing very well with <2 g of proteinuria now.

## 4 DISCUSSION

Our patient's biopsy had features of MGN with diffuse glomerular basement thickening with “spikes” pattern with 2+ IgG4 staining on IF. Extensive subepithelial and intramembranous electron‐dense deposits were seen on biopsy along with extensive foot process effacement in case of nephrotic syndrome with 14 g of proteinuria on 24‐hour urine collection. There were no signs and symptoms suggestive of secondary MGN. He had negative ANA, ANCA, Hepatitis B and C, and had normal complements. Thickened basement membrane with electron‐dense subepithelial deposits containing IgG is hallmark of membranous GN.^2^ It was worth exploring other glomerular causes like membranous‐like GN in differential diagnosis given negative PLA2R and weak IgG staining on biopsy.

Extensive subepithelial and focal intramembranous electron densities were noted on EM but were masked on IF. The fact that IF was only weakly positive for IgG; it could be suggesting that we could be dealing with membranous‐like GN with masked IgG.

Membranous‐like GN also mentioned in literature by the name of membranous glomerulopathy with masked IgG kappa deposits (MGMID) has emerged as a relatively new entity for explaining nephrotic‐range proteinuria and has subepithelial and mesangial deposits by electron microscopy and these deposits are masked on fresh‐frozen IF. Frequently, it has C3 deposits and can be misdiagnosed as C3 glomerulopathy.^3^ Clinical features of MGMID include young women in <40 years of age‐group with known history of the autoimmune process. Spectrum of disease varies from spontaneous remission in some to mild disease to ESRD in others.^3^ In patients with masked immunoglobulin staining of IgG, it can be unmasked using pronase digested biopsy tissue. The patient responded very well to given treatment and the fact that pronase digestion was not available in our hospital, so pronase digested biopsy studies were deferred.

Larsen et al^4^ reported 14 cases of MGMID from review of 10 956 native kidney biopsies over 2 years period. Routine IF was unable to highlight IgG; all patients showed the evidence of subepithelial deposits and 12/14 patients had mesangial deposits. These deposits were unmasked when using pronase digestion. All 14 cases were PLA2R negative when examined using pronase digested section and were thought not to be primary form of membranous glomerulopathy. There were no intramembranous deposits in any of 14 cases of MGMID reported by Larsen et al.^4^ In MGMID, there is little or no IgG staining when IF is performed on fresh‐frozen tissue. Interestingly, on formalin‐fixed paraffin embedded (FFPE) tissue after antigen retrieval reveals positive IF reactivity for IgG‐*kappa*, but not IgG‐*lambda*.^4^

Larsen et al^4^ has nicely delineated the possible reasons for discrepancy between LM, IF, and EM. Frozen unfixed tissue and formalin‐fixed tissue are options for detecting immune deposits on IF. Both have their own merits and demerits. Frozen unfixed tissue is thought to be superior for IF detection of various types of immune‐complex GN (ICGN). Formalin fixation introduces covalent bonds that may conceal antigenic sites, and a protease‐based antigen retrieval step may be necessary to unmask them. Molne et al^5^ suggested that during the process of washing certain antigens are subjected to be lost in an unfixed tissue and fixed tissue can be helpful in such situations.

Miura et al^6^ reported a case in 2008 for IgG negative membranous GN in a lupus patient with no identifiable dense deposits on electron microscopy.

### 4.1 Significance of anti‐PLA2R antibodies

Ronco and Debiec^1^ have summarized results of previous studies regarding the prognostic value of anti‐PLA2R antibodies. Anti‐PLA2R antibodies correlate with proteinuria and disease activity. High titers are correlated to low risk of spontaneous remission and higher risk of the emergence of nephrotic syndrome in non‐nephrotic patients. Diagnostic sensitivity of anti‐PLA2R antibodies was found to be 70.6%, and diagnostic specificity of anti‐PLA2R antibody vs normal and pathological controls was 100% and 94.6%, respectively, as per Radice et al^7^ In 2013, examining 165 biopsies of primary and secondary membranous, Larsen et al^8^ reported sensitivity of 75% and a specificity of 83% for primary membranous glomerulopathy on PLA2R staining. Other causes of positive PLA2R biopsy staining were found to have hepatitis C, sarcoidosis (75%), and neoplasm (25%). Anti‐PLA2R positivity in a patient with MGN should not be considered sufficient to abstain from seeking other causes especially in patients with risk factors for secondary causes or something that just does not fit the clinical picture.^7^

## 5 CONCLUSION

This case report represents primary MGN with positive serum anti‐PLA2R antibodies with 2+ for IgG4 in setting of negative PLA2R on IF with routine fresh‐frozen sections. Patient was treated as primary MGN given positive serum PLA2R autoantibodies and typical biopsy. He responded well to treatment with prednisone. Patient's clinical response also helped to guide the therapy and to confirm the diagnosis of primary membranous GN in this case.

## 6 CONFLICT OF INTEREST STATEMENT

None declared.

## AUTHOR CONTRIBUTION

GK: contributed to writing the whole case report and editing it. GC: contributed to providing pathology slides.
